# Gut microbiota‐derived trimethylamine‐N‐oxide inhibits SIRT1 to regulate SM22α‐mediated smooth muscle cell inflammation and promote atherosclerosis progression

**DOI:** 10.1002/ccs3.70021

**Published:** 2025-06-06

**Authors:** Yajuan Yin, Mei Wei, Xiufang Jiang, Mei Liu, Xiaocui Shi, Xiao Zhang, Le Wang, Gang Liu, Mingqi Zheng, Fangfang Ma

**Affiliations:** ^1^ Department of Cardiology The First Hospital of Hebei Medical University Shijiazhuang Hebei China; ^2^ Department of Medical Affaires The First Hospital of Hebei Medical University Shijiazhuang Hebei China; ^3^ Hebei Key Laboratory of Cardiac Injury Repair Mechanism Study Shijiazhuang Hebei China; ^4^ Hebei Key Laboratory of Heart and Metabolism Shijiazhuang Hebei China; ^5^ Hebei Engineering Research Center of Intelligent Medical Clinical Application Shijiazhuang Hebei China; ^6^ Hebei International Joint Research Center for Structural Heart Disease Shijiazhuang Hebei China

**Keywords:** atherosclerosis, gut microbiota, multi‐omics analysis, SIRT1, SM22α, TMAO

## Abstract

Atherosclerosis (AS) is a prevalent cardiovascular disease, and emerging evidence highlights the critical role of gut microbiota in its development. Trimethylamine‐N‐oxide (TMAO), a metabolite derived from gut microbiota, is thought to promote AS progression by regulating smooth muscle protein 22‐alpha (SM22α)‐mediated inflammation in vascular smooth muscle cells. This study aims to explore the molecular mechanisms of TMAO in AS through multi‐omics analysis, particularly its effects on SIRT1 inhibition and SM22α modulation. 16S ribosomal RNA sequencing revealed an altered gut microbiota composition in AS mice, characterized by increased Bacteroides and decreased Firmicutes. Metabolomics analysis indicated elevated levels of TMAO in AS mice. Transcriptomic data and cell experiments further confirmed that TMAO promotes AS by regulating SM22α‐mediated inflammation via SIRT1 regulation. These findings suggest that TMAO accelerates progression through the SIRT1 and SM22α‐related pathways, offering novel therapeutic targets for AS intervention.

## Graphical abstract

Gut microbiota‐derived metabolite TMAO aggravates atherosclerosis by inhibiting SIRT1, leading to the downregulation of SM22α and promoting smooth muscle cell inflammation via the NF‐κB pathway. This study integrates multi‐omics and in vivo validation to uncover novel therapeutic targets in TMAO–SIRT1–SM22α signaling, offering new insights into microbiota‐driven cardiovascular disease progression.

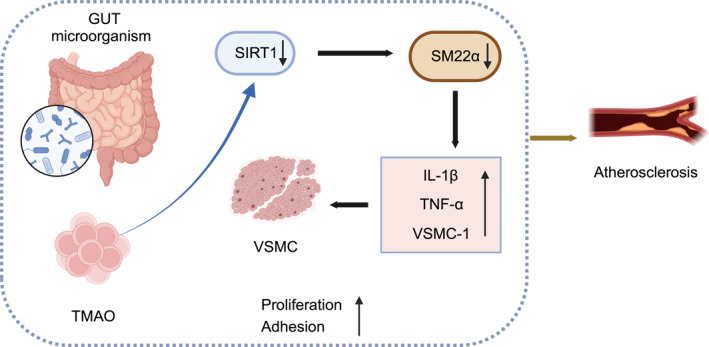

## 1 INTRODUCTION

The incidence of cardiovascular diseases has been steadily rising in recent years, largely driven by changes in modern lifestyle. Among these diseases, atherosclerosis (AS) is particularly critical due to its high prevalence and life‐threatening complications.^1^, ^2^, ^3^ The development of AS is a complex, multifactorial process involving dynamic interactions among various types of cells and bioactive substances. The inflammatory response of smooth muscle cells plays a crucial role in the progression of AS.^2^, ^3^, ^4^, ^5^

Recent research has highlighted the gut microbiota as a key player in the pathogenesis of AS.^6^ Through the production of microbial metabolites, the gut microbiota modulates host physiology and contributes to the progression of cardiovascular disease.^7^ One such metabolite, trimethylamine‐N‐oxide (TMAO), has garnered significant attention for its strong association with cardiovascular risk.^8^, ^9^, ^10^ TMAO is generated via microbial metabolism of dietary choline and carnitine, found abundantly in red meat and egg yolks.^11^ Elevated circulating levels of TMAO have been shown to promote the onset and progression of AS, although the precise cellular mechanisms remain incompletely understood.^12^, ^13^ Current studies increasingly focus on unraveling how TMAO regulates inflammation‐related signaling pathways in smooth muscle cells and how these effects contribute to AS pathogenesis.^14^

SM22α, a cytoskeletal protein specifically expressed in smooth muscle cells (vascular smooth muscle cells [VSMCs]), has been implicated in maintaining vascular homeostasis, influencing cardiovascular disease progression.^15^ It plays critical roles in cytoskeletal stability and cell cycle regulation in both physiological and pathological contexts. In addition, SIRT1, an NAD^+^‐dependent deacetylase, has emerged as a key regulator of TMAO‐induced VSMC inflammation,^16^ offering novel insights into potential intervention targets for AS.^17^

Based on this background, the present study aims to investigate the role of TMAO in SM22α‐mediated VSMC inflammation and AS progression, with a specific focus on SIRT1 signaling. By integrating multi‐omics approaches, including gut microbiota analysis, metabolomics, and transcriptomics, with cellular and animal model validation, we seek to elucidate the underlying molecular mechanisms. Our findings are expected to provide new evidence for the prevention and treatment of AS and to contribute to the development of microbiota‐based strategies for cardiovascular health promotion.

## 2 MATERIALS AND METHODS

### 2.1 Obtaining 16S rRNA sequencing data

To obtain relevant data, the EMBL‐EBI database (https://www.ebi.ac.uk/ena/browser/search) was searched using the keyword “AS,” and phenotype information for all samples from the project with the biological project accession number PRJNA177201 was downloaded. The corresponding 16S rRNA sequencing data were retrieved from the NCBI Sequence Read Archive (SRA) (https://www.ncbi.nlm.nih.gov/sra/). A total of six fecal samples from AS model mice and six from normal control mice were selected for analysis.^18^

### 2.2 Microbial relative abundance analysis

Sample quality was assessed using MultiQC, and host and contaminant sequences were removed using KneadData. The microbial species tree was generated and annotated using GraPhlAn to determine the relative abundance of microbial taxa. Alpha diversity was assessed using the Inverse Simpson index, and beta diversity was analyzed using principal coordinate analysis (PCoA). Differences in microbial abundance and diversity between groups were evaluated using the Wilcoxon rank‐sum test and Welch's *t*‐test. Differential abundance analysis was performed using the edgeR package in R, and the results were visualized through volcano plots and Manhattan plots. Linear discriminant analysis (LDA)and effect size (LEfSe) analyses were applied to identify differentially abundant features, with an LDA score threshold set at 2.0. Higher LDA scores represented greater contributions of specific species to intergroup differences.^19^, ^20^

### 2.3 Microbial functional composition

The FAPROTAX dataset and software (http://www.loucalab.com/archive/FAPROTAX/) were used to predict microbial ecological functions based on taxonomic annotations. Preprocessed QIIME data were analyzed using PICRUSt to infer microbial functional composition. Kyoto Encyclopedia of Genes and Genomes (KEGG) pathways were predicted for each primer set.^21^, ^22^ Statistical analysis and visualization of unstratified results were performed using STAMP software (version 2.1.3). Welch's *t*‐test was used to compare functional group compositions between groups.^23^

### 2.4 Download GEO data

The transcriptome dataset GSE100927, related to AS, was downloaded from the Gene Expression Omnibus (GEO) database (http://www.ncbi.nlm.nih.gov/geo/). Differential gene expression analysis was performed using transcriptomic data from 24 arterial tissue samples from AS patients and 24 control samples. As this dataset was obtained from a publicly available source, no ethical approval or informed consent was required.^24^

### 2.5 Animal model establishment

Twenty‐four healthy C57BL6 mice, weighing approximately 19–21 g and aged 6 weeks (both males and females), were purchased from Beijing Vitonlihua Experimental Animal Technology Co., Ltd. The SIRT1 knock‐in mice were provided by the Nanjing Biomedical Research Institute of Nanjing University. C57BL6 mice were used as the control group and were housed in a pathogen‐free airflow rack under normal air conditions. The mice were kept at a constant temperature of 24–26°C and humidity of 45%–55%. They were provided with high‐temperature sterilized feed and drinking water. The control group was fed a regular diet consisting of 19.6% protein, 4.6% total fat, 4.5% crude fiber, and 14.3 MJ/kg digestible energy (D12102C, Synergia). ApoE^−/−^ AS model mice (NM‐KO‐190565, Nanmo) were provided a high‐fat diet (HFD) consisting of 20% protein, 20% carbohydrates, and 60% fat, which served as the model group (ApoE^−/−^ + HFD) (D12108C, Biotech, Yangzhou).^3^ All animal experiments were approved by the Animal Ethics Committee of our institution and conducted in accordance with the Regulations on the Administration of Laboratory Animals (2021 edition) and the NIH guidelines to minimize animal pain, distress, and discomfort. We strictly adhered to the Declaration of Helsinki.^25^

The mice were divided into three groups: the wild‐type (WT) group consisting of C57BL6 mice, the ApoE^−/−^ + HFD group consisting of ApoE knockout (KO) mice fed with HFD, and the ApoE^−/−^ + Sirt1 + HFD group consisting of ApoE knockout mice with SIRT1 inserted and fed with HFD. The SIRT1‐inserted mice were purchased from Shanghai Namo. All mice were of C57BL6 background, with eight mice in each group.

### 2.6 RNA extraction and sequencing

Three samples of AS mice and arterial tissue samples from mice after fecal microbiota transplantation (FMT) treatment were collected. Total RNA was isolated using Trizol reagent (Invitrogen) with the catalog number 15596026. The concentration and purity of the RNA samples were measured using the Nanodrop 2000 spectrophotometer (1011U, Nanodrop). For future experiments, total RNA samples that meet the following criteria should be considered: RNA integrity number of at least 7.0 and 28S:18S ratio of at least 1.5.

The sequencing library was generated and sequenced by CapitalBio Technology. Each sample uses a total of 5 μg RNA. In summary, the Ribo‐Zero™ Magnetic Kit (MRZE706, Epicenter Technologies) depletes ribosomal RNA (rRNA) from total RNA. The NEB Next Ultra RNA Library Preparation Kit (NEB, #E7775) was utilized for library construction for sequencing on the Illumina platform. Subsequently, the RNA fragment was fragmented into approximately 300 base pair (bp) fragments using the NEB Next First Strand Synthesis Reaction Buffer (5×). The first strand of cDNA was synthesized using reverse transcriptase primers and random primers. The second strand cDNA was synthesized in the second strand synthesis reaction buffer containing dUTP Mix (10×). The repair of cDNA fragment ends involves the addition of a polyA tail and the ligation of sequencing adapters. Following the ligation of Illumina sequencing adapters, the second strand of cDNA was then digested using the USER Enzyme (#M5508, NEB) in order to construct a library that is specific to one strand. The library DNA should be amplified, purified, and enriched using polymerase chain reaction (PCR). Next, the library was assessed using the Agilent 2100 system and quantified with the KAPA Library Quantification Kit (KK4844, KAPA Biosystems). Lastly, paired‐end sequencing was conducted using the NextSeqCN500 (Illumina) sequencer.^26^

### 2.7 Quality control of sequencing data and alignment to a reference genome is performed

The quality of the raw sequencing data was assessed using FastQC software (v0.11.8). Adapter sequences from Illumina platforms and poly(A) tails were removed using Cutadapt (v1.18). Reads containing more than 5% ambiguous bases (N) were eliminated using a custom Perl script. FASTX Toolkit (v0.0.1) was employed to retain reads with base quality scores above 20 in at least 70% of the bases. Paired‐end sequences were repaired using BBMap software. Subsequently, the filtered high‐quality reads were aligned to the mouse reference genome using HiSAT2 (v0.7.12).^26^

### 2.8 Differential gene analysis

Differential gene expression analysis was performed using the “limma” package in R.^27^ Genes related to AS progression were identified based on the criteria of │log_2_FC│ > 0.4 and *p*‐value < 0.05, whereas genes related to gut microbiota alterations were selected using │log_2_FC│ > 0.5 and *p*‐value < 0.05. The results of differential expression were visualized using ggplot2, generating volcano plots to illustrate expression changes.^28^

Venn diagrams were constructed using the “VennDiagram” package in R to identify overlapping differentially expressed genes (DEGs) associated with AS and alternative splicing events.^29^ In addition, target genes associated with the metabolite TMAO were retrieved from the gutMGene database (https://www.genecards.org/).

### 2.9 Weighted gene co‐expression network analysis (WGCNA)

The median absolute deviation (MAD) was first calculated for each gene in the differential expression dataset, and 50% of genes with the smallest MAD values were excluded. Outlier genes and samples were removed using the goodSamplesGenes function from the WGCNA package in R. A scale‐free co‐expression network was then constructed using WGCNA, with the minimum module size set to 40 and sensitivity (deepSplit) set to 3. Modules with a dissimilarity measure less than 0.4 were merged, resulting in the formation of five co‐expression modules. The gray module was considered to contain genes that did not belong to any specific module.

Subsequently, the correlation between module eigengenes and experimental groups was evaluated using the Pearson correlation test (*p* < 0.05). Modules significantly associated with AS, particularly those containing BRAF‐related genes, were selected for further analysis.^30^

### 2.10 VSMCs culture

Eight male C57BL/6 mice (19–21 g) were used in this study. Following euthanasia, the aortas were carefully excised and rinsed three times with phosphate‐buffered saline (PBS) to remove residual blood. The adventitia and perivascular adipose tissue were removed in a biosafety cabinet. The cleaned aortas were cut into 1–2 mm segments and incubated in 1% collagenase solution (17104019, Thermo Gibco™) at 37°C for at least 2 h. The resulting digests were filtered through a 40 μm cell strainer, and cells were collected by centrifugation at 1000 rpm for 5 min. The supernatant was discarded, and the cells were resuspended in pre‐warmed low‐glucose Dulbecco's Modified Eagle Medium (DMEM) (11965092, Gibco) supplemented with 20% FBS. The cells were then seeded onto collagen‐coated dishes and incubated at 37°C with 5% CO_2_ in a Heracell™ Vios 160i CO_2_ incubator (51033770, Thermo Scientific™, Germany). When cell confluence reached 70%–80%, the cells were passaged using 0.25% trypsin‐EDTA for 5–10 min. The culture medium was replaced every 2–3 days.^31^, ^32^

VSMCs were identified by immunostaining for α‐smooth muscle actin (α‐SMA, ab7817, Abcam), a specific marker for VSMCs.^33^

Cell experiments were conducted when cells reached 80%–90% confluence after 3–6 passages. The cells were treated with TMAO at concentrations of 0, 150, 300, 600, and 900 μmol/L for 24 h or exposed to 600 μmol/L TMAO (HY‐114202, MCE) for varying time intervals (0, 4, 12, and 24 h).^34^ Untreated VSMCs served as the control group.

### 2.11 Cell infection

To perform overexpression or knockdown of Sirt1 and SM22α, VSMCs were seeded into 6‐well plates and cultured until they reached 70%–90% confluence. A lentiviral solution (MOI = 10, titer ∼5 × 10^6^ TU/mL) containing 5 μg/mL polybrene (TR‐1003, Merck) was added. After 4 h of transduction, an equal volume of medium was added to dilute the polybrene. After 24 h, the culture medium was replaced with fresh medium. At 48 h post‐transduction, luciferase reporter activity was used to assess transfection, and stably transfected cells were selected using 1 μg/mL puromycin (A1113803, Thermo Fisher).^35^, ^36^, ^37^

Infection efficiency was verified by real‐time quantitative reverse transcription PCR (RT‐qPCR), and sequences showing efficient modulation were used for subsequent experiments.^38^ Each experiment was repeated three times. The recombinant lentiviruses and plasmids were constructed and provided by Shanghai GenePharma Co., Ltd.

### 2.12 Co‐culture and adhesion experiments

For co‐culture assays, primary bone marrow‐derived macrophages (BMDMs) were obtained from CP‐M141 (Pricella) and seeded at a density of 3 × 10^6^ cells/mL. BMDMs were differentiated in DMEM (11965092, Gibco) containing 10% FBS and 20 ng/mL M‐CSF for 7 days. All cells were regularly tested to ensure absence of Mycoplasma contamination (C0301S, Beyotime).

BMDMs were labeled with BCECF‐AM (10 μM, S1006, Beyotime) at 37°C in DMEM for 1 h, followed by washing and centrifugation. VSMCs pre‐treated with 600 μmol/L TMAO for 24 h in 12‐well plates were washed twice with PBS. Labeled BMDMs (2 × 10^6^ cells/mL) were then seeded onto the VSMC monolayer and co‐cultured for 1 h. Non‐adherent BMDMs were removed by PBS washing, and the adhesive capacity of cells was visualized using a fluorescence microscope.^34^

### 2.13 EdU staining

VSMCs at 50%–70% confluence were used for EdU incorporation assays. After discarding the culture medium, cells were washed twice with PBS and then incubated overnight with 10 mM EdU solution (C10418, Thermo Fisher) diluted in culture medium. After incubation, the medium was removed, and cells were fixed with 50 μL of 4% paraformaldehyde (PFA) for 20 min at room temperature.

Residual fixative was neutralized with 50 μL of 2 mg/mL glycine solution for 5 min. The cells were washed twice with 0.1 mL of 3% bovine serum albumin in PBS per well. Then, 0.1 mL of 0.5% Triton X‐100 in PBS was added and incubated for 20 min at room temperature. Finally, the reaction solution was applied and incubated for 30 min at room temperature. The proportion of proliferating cells was determined by assessing EdU fluorescence under a fluorescence microscope.^39^

### 2.14 Western blot

Total protein was extracted from cells and tissues. Cells and tissues were digested and collected using trypsin (T4799‐5G, Sigma‐Aldrich). Cell lysis was performed using an enhanced radioimmunoprecipitation assay buffer (RIPA) lysis buffer (AR0108, Wuhan Bosideng Co., Ltd.) containing a protease inhibitor. The protein concentration was determined using the bicinchoninic acid assay protein quantification kit (AR1189, Wuhan Bosideng Co., Ltd.). Proteins were separated by SDS‐PAGE, followed by transfer onto a polyvinylidene difluoride membrane. The membrane was blocked with 5% BSA (Sigma‐Aldrich; 9048‐46‐8) for 1 h at room temperature. A diluted primary antibody (Table S1) was applied and incubated overnight at 4°C. The membrane was washed three times with phosphate‐buffered saline with tween‐20 (PBST) (3 × 5 min), followed by incubation with an Anti‐Mouse‐HRP secondary antibody (7076, 1/5000; CST) for 1 h at room temperature. Afterward, the membrane was washed three times with PBST (3 × 5 min). The PBST was discarded, and an appropriate amount of ECL working solution (Omt‐01, Beijing Aomijiade Pharmaceutical Technology Co., Ltd.) was added. The membrane was incubated at room temperature for 1 min, and excess ECL reagent was removed. The membrane was sealed with cling film and exposed to X‐ray film in a dark box for 5–10 min before development and fixation. The grayscale values of protein bands in Western blot images were quantified using ImageJ analysis software, with β‐actin used as an internal reference.^40^, ^41^

### 2.15 LC‐MS is the combination of liquid chromatography and mass spectrometry

Mouse fecal samples were collected and transferred into individual 1.5 mL polypropylene tubes, with 300 μL per sample. Each sample was mixed with 900 μL of 80% methanol (67‐56‐1, Sigma‐Aldrich) and 0.1% formic acid (64‐18‐6, Sigma‐Aldrich). The mixture was vortexed for 2 min and centrifuged at 12,000 g for 10 min. The supernatant was transferred into vials for automated sampling.

Fecal metabolomics analysis was performed using a LC20 ultra‐high‐performance liquid chromatograph (SHIMADZU) coupled with a Triple TOF‐6600 mass spectrometer (AB Sciex). Chromatographic separation was conducted using a Waters ACQUITY UPLC HSS T3 C18 column (100 × 2.1 mm, 1.8 μm) maintained at 40°C. The flow rate was set at 0.4 mL/min, and the mobile phase consisted of an aqueous acetonitrile solution containing 0.1% formic acid (CAS No. 75‐05‐8, Sigma‐Aldrich). The gradient elution program for mobile phase B was as follows: 5% (0–11 min), 90% (11–12 min), and 5% (12.1–14 min). The rinse solution was introduced directly into the mass spectrometer without fragmentation.^42^

Mass spectrometry was performed in both positive and negative ion modes, with an ionization voltage of 5500 V, a capillary temperature of 550°C, a spray gas flow rate of 50 psi, and an auxiliary heating gas flow rate of 60 psi. Data analysis was carried out using Orthogonal Partial Least Squares Discriminant Analysis and permutation testing (100 permutations) to prevent overfitting. Metabolites with VIP scores greater than 1 and *p*‐values less than 0.05 were classified as differential metabolites (DMs). The final DMs were selected based on a fold change ≥2 or ≤0.5 and a *p*‐value < 0.05 in Student's *t*‐test, accounting for single‐variable analysis. Relevant metabolic pathways were identified using MetaboAnalyst (Version 5.0).^43^

### 2.16 Gas chromatography‐mass spectrometry method (GC‐MS)

Mouse serum was collected, and 10 μL of ultrapure water was added to every 1 mL of serum. The mixture was thoroughly mixed by shaking and allowed to stand for 30 min. The sample was then centrifuged at 4°C and 12,000 rpm for 20 min. After centrifugation, the supernatant was aspirated and filtered through a 0.22 μm hydrophilic membrane before being transferred to a gas bottle for measurement.

A deactivated bonded polyethylene glycol wax column 30M chromatographic column (I.D. 0.32 mm, 5 μm, Agilent) was used for separation. Both the injector and detector temperatures were set to 250°C. The temperature program was as follows: the initial temperature was maintained at 50°C for 3 min, then increased to 120°C at a rate of 6°C per minute, and held for 0.5 min. The temperature was further increased to 220°C at 6°C per minute and maintained for 5 min. The flow rates for nitrogen, hydrogen, and air were 3, 47, and 400 mL/min, respectively, with a split ratio of 1:3. A sample volume of 1.0 μL was injected. Quantification was performed using the external standard method. The content of TMA and TMAO in the sample was determined by calculating the ratio of the peak area of the standard sample to the peak area of the sample.^44^, ^45^

### 2.17 ELISA

Mouse serum or cell lysate samples were collected, and the expression levels of IL‐1β (ab197742, Abcam) and TNF‐α (ab208348, Abcam) were measured using the enzyme‐linked immunosorbent assay (ELISA) kit (ab171523, Abcam).

To prepare cell lysates, the cells were washed with PBS and collected, followed by centrifugation to pellet the cells. The supernatant was discarded, and the cell pellet was treated with RIPA lysis buffer (AR0108, Wuhan Bosideng Co., Ltd.) and incubated on ice. High‐speed centrifugation was performed to remove cellular debris, and the supernatant was collected as the cell lysate. The protein concentration was determined using the AR1189 Protein Assay Kit (Wuhan Boshite Co., Ltd.).

For ELISA, the target antigen was immobilized on a solid phase carrier, non‐specific binding sites were blocked, and the test sample was incubated with the antigen. An enzyme‐labeled secondary antibody was added and allowed to react with the corresponding substrate. The enzymatic product was measured for absorbance using a spectrophotometer, which allowed for the quantitative or qualitative determination of antibodies or antigens in the sample.^46^

### 2.18 Detection of blood lipids and oxidative stress levels

To measure triglyceride (TG) levels, a TG content detection kit (BC0625, Solarbio) was used on mouse serum, following the manufacturer's instructions. Additionally, the malondialdehyde (MDA) detection kit (Solarbio, BC0025) was used to assess MDA levels in both mouse arterial tissue homogenate and serum.

For blood lipid testing, approximately 5–10 mL of venous blood was collected from the mice into an anticoagulant‐free tube (YA1461, Solarbio). The blood sample was left at room temperature (20–25°C) for 30 min to allow coagulation. Afterward, the blood sample was centrifuged at 12,000 g for 10 min. The TG levels were then measured using the TG content detection reagent kit.

For oxidative stress measurement, approximately 5 mL of venous blood was collected using an anticoagulant tube containing EDTA (YA1461, Solarbio). The blood was centrifuged at 12,000 g for 10 min, and the MDA levels were subsequently measured using the Micro MDA detection kit.^47^

### 2.19 Oil red O staining

The mice were euthanized 12 weeks after HFD treatment. After perfusing the left ventricle with physiological saline and 4% PFA, the aorta was excised from the ascending aorta down to the iliac bifurcation. The aorta was then stained with a 0.3% Oil Red O solution at room temperature to visualize both the surface and cross‐section. After a 5‐min incubation, the aorta was washed with 60% isopropanol for 10 s. The samples were subsequently hydrated in PBS and observed under a microscope for imaging.

The hearts of the mice underwent dehydration using a sucrose gradient, followed by embedding in OCT compound and sectioning into 10 μm thick slices from the aortic root. The slices were hydrated in PBS, stained with 0.3% Oil Red O, and then dehydrated using 95% ethanol and xylene. Finally, the sections were mounted and examined under an optical microscope (Olympus, CX43). Results were subjected to semi‐quantitative analysis using Image Pro Plus software.^25^

### 2.20 Fecal microbiota transplantation (FMT)

Feces were collected from normal C57BL6 WT donor mice and combined. The fecal matter was diluted with cold PBS at a ratio of 100 mg of feces per 1 mL of buffer. The mixture was thoroughly mixed for 10 min to form a paste‐like consistency. The sample was vortexed for 1 min and centrifuged at 800 g for 5 min. The supernatant was collected, mixed with an equal volume of 80% glycerol‐PBS as cryoprotectant, aliquoted, and stored at −20°C until transplantation.

To reduce the bacterial burden in recipient mice, an antibiotic mixture was administered via oral gavage once daily for 7 consecutive days. The antibiotic cocktail included vancomycin (50 mg/kg), neomycin (100 mg/kg), metronidazole (100 mg/kg), and amphotericin B (1 mg/kg). Ampicillin (1 g/L) was added to drinking water.^48^ Following the antibiotic regimen, ApoE^−/−^ AS receptor mice on a HFD were orally administered 1 mL of donor gut microbiota supernatant every other day for 2 weeks.^49^ Mice in the vehicle control group received an equal volume of 80% glycerol‐PBS (without fecal content) using the same schedule to eliminate potential effects from the cryoprotectant solution.

### 2.21 MTT assay for cell viability

Cells were inoculated at a density of 1 × 10^4^ cells per well in a 96‐well plate using culture medium and incubated for 24 h. Cell viability was assessed by incubating cells from various treatment groups with culture medium supplemented with 0.5 mg/mL 3‐(4,5‐dimethylthiazol‐2‐yl)‐2,5‐diphenyltetrazolium bromide (MTT) (Sigma‐Aldrich, product code: 298‐93‐1) for 4 h. The absorbance was measured at 570 nm.^50^

### 2.22 RT‐qPCR

Cells or tissues were lysed, and total RNA was extracted using Trizol Reagent (10296010, Invitrogen, Thermo Fisher). RNA quantification and quality assessment were performed using UV‐visible spectrophotometry (ND‐1000, Nanodrop, Thermo Fisher). Reverse transcription was carried out using the PrimeScript™ RT‐qPCR Kit (RR086A, TaKaRa). RT‐qPCR was conducted using SYBR Premix Ex Taq™ (DRR820A, TaKaRa) on the LightCycler 480 system (Roche Diagnostics). β‐Actin was used as the internal reference for mRNA expression. The amplification primers were designed and provided by Shanghai General Bioscience Co., Ltd. The primer sequences are listed in Table S2. The fold change in gene expression between experimental and control groups was determined using the 2^−ΔΔCt^ method, with ΔCt calculated as Ct target gene—Ct reference gene.^51^

### 2.23 Statistical analysis

Statistical analysis was performed using R software (version 4.2.1), and RStudio (version 4.2.1) was used for compilation. Data processing was done using Perl (version 5.30.0). Cytoscape (version 3.7.2) was used for network analysis, and statistical analyses were conducted using SPSS (version 21.0, IBM SPSS Statistics). Measurement data are presented as mean ± standard deviation.

Comparisons between two groups were performed using an independent sample *t*‐test, and comparisons between different time points were made using repeated measures analysis of variance, followed by Bonferroni correction for post‐hoc testing. A *p*‐value < 0.05 was considered statistically significant.

## 3 RESULT

### 3.1 Differential gut microbiota profiles in AS: A comparative study between AS model mice and normal mice

**FIGURE 1:**
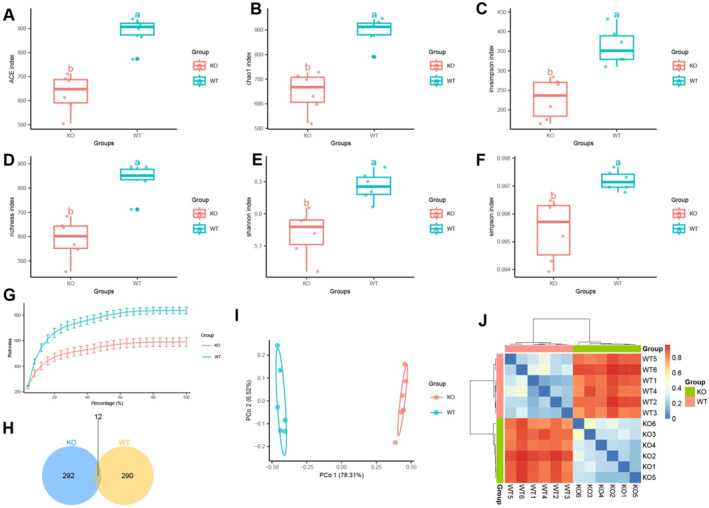
Analysis of intestinal microbial diversity in fecal samples of KO and wild‐type (WT) mice. (A–F) Alpha diversity analysis of intestinal microbiota in KO and WT mice, with (A) representing the ACE index, (B) representing the Chao1 index, (C) representing the Invsimpson index, (D) representing the Richness index, (E) representing the Shannon index, and (F) representing the Simpson index. (G) Rarefaction curve of intestinal microbial alpha diversity in KO and WT mice. (H) Venn diagram showing the intersection of amplicon sequence variants in intestinal microbiota of KO and WT mice. (I) Principal coordinates analysis plot showing the Beta diversity of intestinal microbiota in KO and WT mice. (J) Heatmap of distance matrix based on Beta diversity of intestinal microbiota in KO and WT mice; a and b indicate statistically differences between the two groups (*p* < 0.05); KO group, *n* = 6; WT group, *n* = 6.

To investigate the molecular mechanisms through which the gut microbiota influences AS we initially analyzed the disparities in gut microbiota between AS mice and normal mice; we obtained 16S rRNA sequencing data for fecal samples from six mice in the KO group and six mice in the WT group, all of the same age, using the SRA database. Among them, the KO group comprises ApoE^−/−^ knock‐out mice fed a HFD, serving as the AS model mice. The WT group consists of C57 mice. Initially, we assessed the variations in species composition between the two groups using alpha diversity analysis. Alpha diversity is employed to assess the species composition in a given sample, encompassing species richness and abundance. Frequently employed algorithms for biodiversity analysis include ACE, Chao1, Invsimpson, Richness, Shannon, and Simpson indices. The results indicate that the ACE, Chao1, Invsimpson, Richness, Shannon, and Simpson indices of the WT group were higher when compared to the KO group (Figure 1A–F). The analysis of α‐rarefaction curves revealed that with an increase in the percentage of sample size, the richness of both the WT and KO groups increased at a diminishing rate until it reached a saturation point (Figure 1G). This finding suggests that the diversity and richness of gut microbiota in the WT group are higher compared to the KO group.

Moreover, we conducted a Venn analysis on the Amplicon Sequence Variants (ASVs) that were enriched in the WT group and knockout (KO) group, respectively. The results indicated that there were 290 ASVs in the WT group which differed from those in the KO group, whereas the KO group had 292 ASVs in its species composition that differed from the WT group (Figure 1H). Beta diversity is utilized to examine the variations in species composition among different communities. Clear separation between the two groups of samples was observed through PCoA (CoA) (Figure 1I). Moreover, the beta diversity distance matrix was calculated using the Bray–Curtis method. A heat map (Figure 1J) was then plotted, illustrating the Bray–Curtis distance. The results indicate a distinct separation and difference between the two groups of samples.

Next, we examined the variations in the abundance of gut microbiota between the WT and KO groups. The results demonstrated differences in ASVs between the knockout (KO) group and the WT group, as determined by volcano plots, heatmaps, and Manhattan plots. Compared to the KO group, the WT group exhibited 141 decreased ASVs and 166 increased ASVs (Figure S1A–C).

To conclude, there are notable disparities in the diversity and abundance of gut microbiota between mice with AS and those without the condition.

### 3.2 Distinct gut microbiota composition in AS mice and normal mice: A comprehensive analysis across multiple taxonomic levels

To further investigate the differences in species composition of gut microbiota between AS and normal mice, we analyzed the gut microbiota at the phylum, class, order, family, and genus levels in both the KO and WT groups. Additionally, we generated a dendrogram and a stacked bar chart.

**FIGURE 2:**
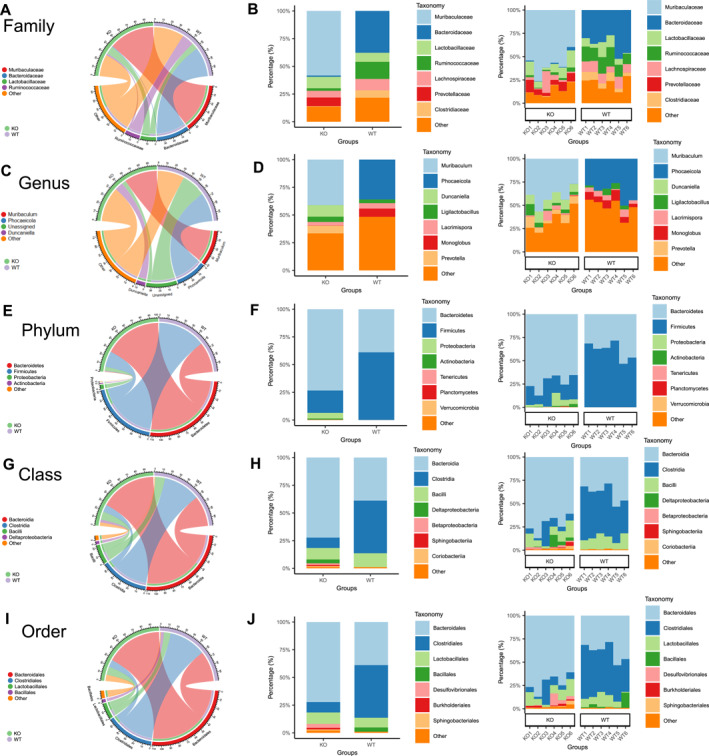
Differential analysis of microbial composition at different levels between KO and wild‐type (WT) mice. (A) Chord diagram showing the family‐level abundance of intestinal microbiota in KO and WT mice. (B) Stacked bar chart showing the proportional abundance of intestinal microbiota at the family level in KO and WT mice. (C) Chord diagram showing the genus‐level abundance of intestinal microbiota in KO and WT mice. (D) Stacked bar chart showing the proportional abundance of intestinal microbiota at the genus level in KO and WT mice. (E, F) Chord diagram and stacked bar chart showing the phylum‐level abundance of intestinal microbiota in KO and WT mice. (G, H) Chord diagram and stacked bar chart showing the class‐level abundance of intestinal microbiota in KO and WT mice. (I, J) Chord diagram and stacked bar chart showing the order‐level abundance of intestinal microbiota in KO and WT mice; KO group, *n* = 6; WT group, *n* = 6.

The results indicated that, at the phylum level, the predominant microorganisms in both groups were found in the families Bacteroidaceae and Muribaculaceae. Within the KO group, Muribaculaceae exhibits higher abundance than that in the WT group, whereas Bacteroidaceae and Ruminococcaceae show higher abundance in the WT group compared to the KO group (Figure 2A,B). At the genus level, the two groups of microorganisms are primarily distributed among the genera Muribaculum, Duncaniella, and Phocaeicola. The KO group exhibited a higher abundance of Muribaculum and Duncaniella compared to the WT group, whereas Phocaeicola was more abundant in the WT group (Figure 2C,D).

At the phylum level, microorganisms in the two groups are mainly found in the species Firmicutes and Bacteroidetes. The KO group showed a notably higher abundance of Bacteroidetes compared to the WT group (Figure 2E,F). At the class level, these two groups of microorganisms are predominantly found in the orders Clostridia, Bacteroidia, and Bacilli. The abundance of Bacteroides is higher in the KO group compared to the WT group, whereas Clostridia is more abundant in the WT group than in the KO group (Figure 2G,H). At the taxonomic order level, the two groups of microorganisms are mainly found in the taxonomic orders Clostridiales, Bacteroidales, and Lactobacillales. The number of Bacteroidales was higher in the KO group compared to the WT group, whereas the WT group had a greater abundance of Clostridiales in comparison to the KO group (Figure 2I,J).

To explore the variations in the species composition of gut microbiota between the knockout (KO) and WT groups, we performed a LEfSe analysis using a significance threshold of LDA score (log 10 > 3) as the criteria for identifying differences. The results indicated that the relative abundance of Bacteroidales, Bacteroidia, Bacteroidetes, Muribaculum, Duncaniella, and Muribaculaceae in the KO group fecal samples was higher compared to the WT group. Conversely, the relative abundance of Ruminococcaceae, Clostridiales, Clostridia, and Firmicutes was higher in the WT group than in the KO group (Figure S2A,B).

The results presented above indicate variations in the composition of intestinal bacteria between AS mice and normal mice, suggesting a role of the gut microbiota in the pathogenesis of AS.

### 3.3 Functional discrepancies in gut microbiota between AS mice and normal mice: A KEGG pathway analysis

**FIGURE 3:**
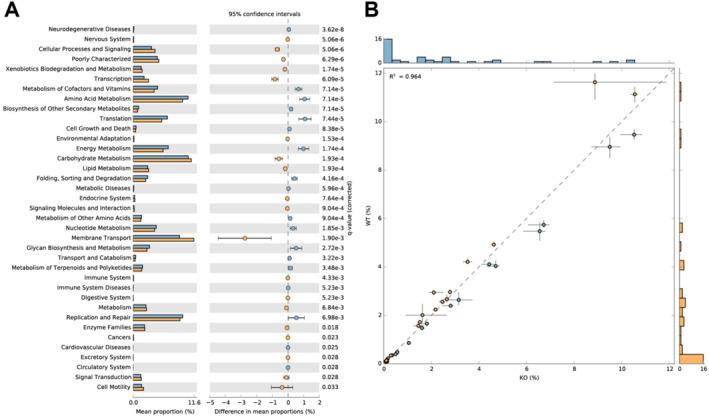
Functional enrichment analysis of differential intestinal microbiota between wild‐type (WT) and KO groups. (A) Bar plot showing the functional enrichment analysis of intestinal microbiota in the WT and KO groups. (B) Scatter plot showing the functional enrichment analysis of intestinal microbiota in the WT and KO groups; KO group, *n* = 6; WT group, *n* = 6.

To examine the variations in the functional composition of the gut microbiota between the KO and WT groups, we employed the PIParkinsonismUSt2 software to analyze the KEGG enrichment of the gut microbiota based on the abundance data of genera. We used the STAMP software to visually represent the functional composition of the gut microbiota. The results demonstrated that there were 13 statistically enriched signaling pathways in the differential gut microbiota between the knockout (KO) group and the wildtype (WT) group, with a *p*‐value less than 0.05. These pathways encompassed transcription, co‐factors and vitamin metabolism, amino acid metabolism, translation, energy metabolism, carbohydrate metabolism, lipid metabolism, nucleotide metabolism, membrane transport, polysaccharide biosynthesis and metabolism, replication and repair, signal transduction, and cell motility (Figure 3A,B). It is worth noting that membrane transport and amino acid metabolism exhibited the highest significance level. Previous studies have demonstrated the crucial role of amino acids in specific biological processes associated with arterial inflammation and hardening.

Moreover, it is suggested that the gut microbiota may influence the progression of AS by affecting amino acid metabolism.^52^ This result is consistent with our analysis results. Furthermore, the substantial disparities in membrane transport could imply the notable alterations in competition for and interaction of nutrients between the microbiota and host cells.

In summary, the results above indicate alterations in gut microbiota function between AS mice and normal mice. These alterations may play a role in the development and progression of AS by actively participating in the inflammatory pathway.

### 3.4 Differential metabolomic analysis reveals the role of gut microbiota‐driven TMAO in AS development

**FIGURE 4:**
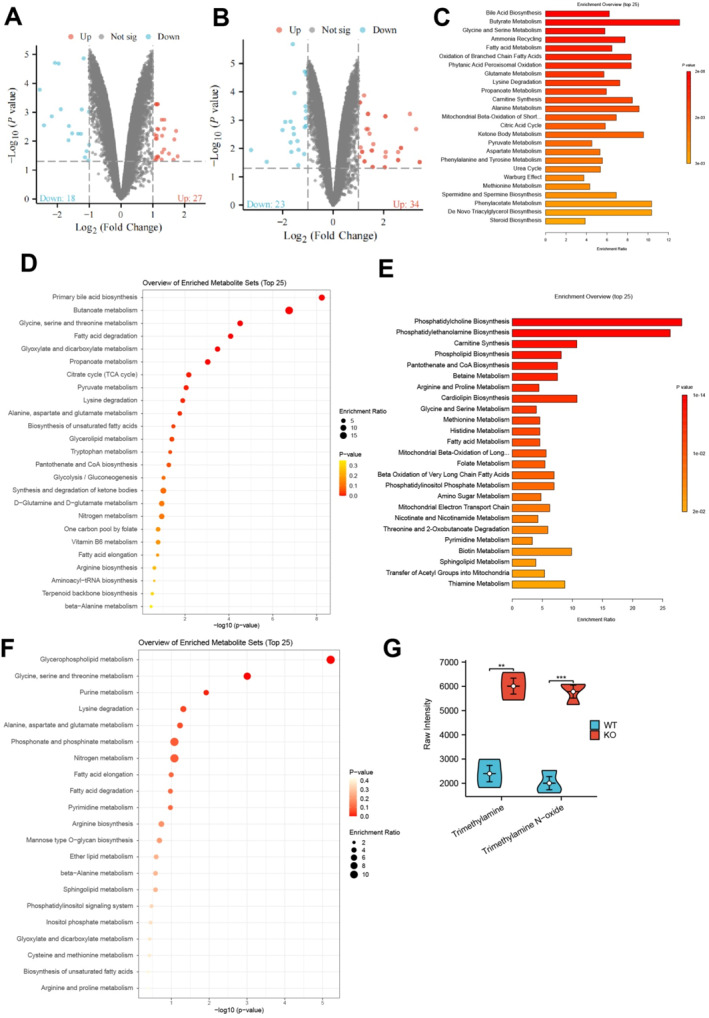
Differential metabolites (DMs) between KO and wild‐type (WT) mice in metabolomics analysis. (A) Volcano plot of DMs in negative ion mode, with red dots representing upregulated metabolites in the KO group, blue dots representing downregulated metabolites in the KO group, and gray dots indicating metabolites with no difference. (B) Volcano plot of DMs in positive ion mode, with red dots representing upregulated metabolites in the KO group, blue dots representing downregulated metabolites in the KO group, and gray dots indicating metabolites with no difference. (C, D) Functional enrichment analysis of DMs in negative ion mode using the MetaboAnalyst database, with (C) showing the SMPDB results and (D) showing the Kyoto encyclopedia of genes and genomes (KEGG) results. (E, F) Functional enrichment analysis of DMs in positive ion mode using the MetaboAnalyst database, with (E) showing the SMPDB results and (F) showing the KEGG results. (G) Differences in the levels of the metabolites TMA and trimethylamine‐N‐oxide in the serum of KO and WT mice in positive ion mode; KO group, *n* = 6; WT group, *n* = 6; *p < 0.05, **p < 0.01, ***p < 0.001, analyzed using one‐way analysis of variance and *t*‐test.

Metabolomics is a technique used to comprehensively describe all low molecular weight metabolites in biological samples.^53^ To gain a comprehensive understanding of the precise molecular mechanisms through which the gut microbiota influences the advancement of AS, we performed a comparative analysis of metabolic products in AS mice and healthy mice. We obtained fecal samples from a total of 6 AS mice and 6 normal mice. Subsequently, we conducted untargeted metabolomics analysis by employing liquid chromatography‐mass spectrometry (LC‐MS) technology. Following quality control analysis, we employed multiple analyses and *t*‐tests to identify DMs. The volcano plot demonstrates the disparities in filtering outcomes between positive ion mode and negative ion mode. A total of 45 different metabolites were identified in negative ion mode, consisting of 27 upregulated and 18 downregulated compounds (Figure 4A). Fifty‐seven distinct metabolites were identified in positive ion mode, with 34 showing upregulation and 23 showing downregulation (Figure 4B). We performed a cluster analysis on the DMs using hierarchical clustering and the Euclidean distance metric.

We then performed functional enrichment analysis on the DMs obtained from the positive and negative ion modes using the MetaboAnalyst 5.0 database. The results of the analysis conducted on the SMPDB database revealed that the DMs were primarily enriched in pathways including fatty acid metabolism, branched‐chain fatty acid oxidation, inositol phosphate oxidation, glutamate metabolism, alanine metabolism, ketone body metabolism, phenylalanine metabolism, and tyrosine metabolism (Figure 4C). In the analysis using the KEGG database, DMs are primarily enriched in pathways, including primary bile acid biosynthesis, butanoate metabolism, and glycine, serine, and threonine metabolism (Figure 4D). Based on the enrichment analysis results from two databases, we have identified the bile acid metabolism pathway and the branched‐chain fatty acid metabolism as potentially crucial pathways associated with the DMs in the negative ion mode. The disparities in metabolic pathways correspond to the variations in lipid accumulation pathways between AS and normal mice.

Furthermore, when analyzing the SMPDB database in the positive ion mode, it was observed that differentially metabolized compounds are primarily enriched in pathways related to the synthesis of phosphatidylcholine, phosphatidylethanolamine, carnitine, and myocardial phospholipids (Figure 4E). The KEGG database analysis revealed that the differentially regulated metabolites were primarily enriched in pathways such as glycerophospholipid metabolism, glycine, serine, and threonine metabolism, as well as purine metabolism (Figure 4F). Based on the enrichment analysis results from two databases, we observed that the pathways involved in phosphatidylcholine synthesis and carnitine synthesis might serve as prominent pathways related to DMs in the positive ion mode.

The literature review reveals that the gut microbiota produces TMA during the breakdown of carnitine and choline. Subsequently, TMA is oxidized to TMAO in the liver.^9^ Recent studies have identified elevated levels of TMAO as an independent risk factor for cardiovascular diseases. TMAO contributes to cholesterol deposition on arterial walls, increases inflammatory responses, and enhances the formation of atherosclerotic plaques.^54^ In addition, we assessed the serum metabolic levels of TMA and its oxidative derivative TMAO in mice. The results showed that serum TMA levels were significantly elevated in the KO group compared to the WT group, accompanied by a corresponding increase in TMAO levels (Figure 4G).

In conclusion, TMAO may serve as a metabolite influenced by the gut microbiota during the development of AS.

### 3.5 Alleviation of AS through FMT: A reduction in TMAO levels and atherosclerotic inflammation

FMT, a method used to directly manipulate the gut microbiota, has been extensively employed in diseases associated with gut microbiota dysbiosis.^55^ To examine the influence of gut microbiota on the occurrence and progression of AS, along with the metabolic profile of key metabolites, we conducted FMT on mice with AS.

**FIGURE 5:**
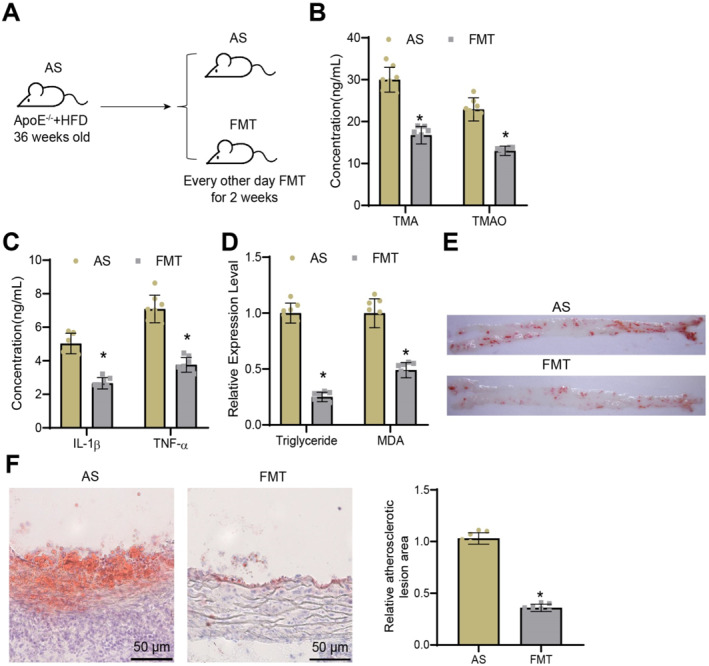
Effects of fecal microbiota transplantation (FMT) treatment on disease progression and trimethylamine‐N‐oxide TMAO in atherosclerosis (AS). (A) Schematic diagram of FMT treatment in mice. (B) Metabolite levels of TMA and TMAO in serum. (C) Levels of inflammatory factors in serum measured by ELISA. (D) Levels of serum triglyceride and malondialdehyde in each group of mice. (E) Representative images of atherosclerotic lesions in the aorta of each group of mice stained with oil red O. (F) Quantification of aortic lesion levels in the root section stained with oil red O, with representative images on the left and quantification chart on the right (scale bar = 50 μm); * indicates *p* < 0.05 compared to the AS group; *n* = 8 mice in the animal experiment.

In summary, we initially subjected the mice to 1 week of antibiotic treatment to reduce the gut bacterial load. After this pretreatment, FMT was conducted every other day for 2 weeks by oral gavage of 1 mL of fecal microbiota supernatant derived from healthy C57BL6 mice. Control mice received an equal volume of 80% glycerol‐PBS solution to account for the vehicle effect. Samples were collected for analysis after completion of the FMT course. The mice were euthanized 2 weeks after successful FMT treatment, and pertinent samples were examined (Figure 5A).

We first analyzed the levels of TMA and TMAO in serum using gas chromatography‐mass spectrometry (GC‐MS). The results demonstrated a reduction in the metabolic levels of TMA and TMAO in the FMT group compared to the AS group (Figure 5B). Previous studies have demonstrated an association between TMAO and the pathways of AS and inflammation. Hence, we employed ELISA kits to quantify the concentrations of inflammatory cytokines TNF‐α and IL‐1β, which are correlated with inflammation in atherosclerotic smooth muscle cells.^56^ The results demonstrated a noteworthy reduction in levels of inflammatory cytokines in the FMT group as compared to the AS group (Figure 5C).

According to relevant literature, TG and MDA are oxidative markers that have a close association with AS. These markers reflect the complex relationship between lipid metabolism, oxidative damage, and atherosclerotic inflammation.^57^ In addition, we quantified TG levels and measured the oxidative stress marker MDA. Moreover, we visualized the atherosclerotic lesions in the mouse aorta through oil‐red staining. Compared to the AS group, the FMT group displayed lower serum levels of TG and MDA, along with reduced numbers and sizes of aortic plaques, and decreased area of aortic root tissue lesions (Figure 5D,F).

In conclusion, FMT treatment has the potential to substantially decrease the levels of TMAO in mouse serum, thus effectively improving the condition of AS.

### 3.6 Elucidating the molecular mechanisms of TMAO in AS progression: The central role of Sirt1 and gene expression modulation

**FIGURE 6:**
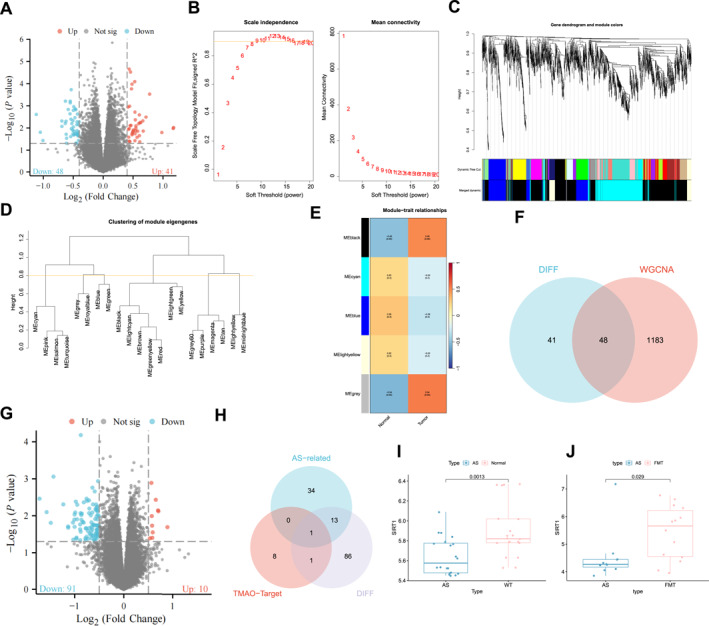
Key genes selected by transcriptomic analysis for the influence of trimethylamine‐N‐oxide TMAO on the progression of atherosclerosis (AS). (A) Volcano plot showing differentially expressed genes (DEGs) in arterial tissues between Normal mice and AS group based on transcriptomic data, including 24 normal and 24 AS samples. Red represents upregulated genes, blue represents downregulated genes, and gray represents genes with no difference. (B) Scale independence, average connectivity, and scale‐free topological structure to determine the weight value *β* that satisfies the scale‐free network law, *β* = 9. (C) Cluster dendrogram and trait heatmap of transcriptomic data from normal and AS mice samples. (D) Cluster dendrogram for sample selection. (E) Correlation coefficients and correlation analysis between different gene modules and AS. (F) Intersection of DEGs between wild‐type (WT) and AS groups and disease‐related genes obtained from weighted gene co‐expression network analysis. (G) Volcano plot showing differential gene expression in arterial tissues between AS group and fecal microbiota transplantation (FMT) group, including 3 AS samples and 3 FMT treatment samples. Red represents upregulated genes, blue represents downregulated genes, and gray represents genes with no difference. (H) Intersection of target genes of TMAO, differential regulation of gut microbiota, and DEGs related to AS progression. (I) Differential expression of Sirt1 in WT group and AS group mice. AS group, *n* = 24; WT group, *n* = 24. (J) Differential expression of Sirt1 in AS group and FMT group mice. AS group, *n* = 3; FMT group, *n* = 3.

In order to examine the precise molecular mechanisms underlying the effects of TMAO, a metabolite of gut microbiota, on AS, we retrieved the AS‐related chip GSE100927 from the GEO database and randomly chose 24 normal samples and 24 AS samples. Differential gene expression analysis revealed 99 DEGs in the arterial tissues of normal mice and mice with AS, encompassing 48 genes that were downregulated and 41 genes that were upregulated (Figure 6A). Subsequently, we employed the WGCNA algorithm to build a co‐expression network encompassing all genes. We carefully selected a suitable threshold to strengthen the gene correlations, ensuring that the resulting network adheres to the characteristic traits of a scale‐free network. The analysis results indicate that the chosen optimal threshold is 9 (Figure 6B). We constructed gene co‐expression networks using a threshold of 9. To perform hierarchical clustering of genes in the colorectal cancer dataset, a dissimilarity matrix was utilized, resulting in the generation of a clustering dendrogram (Figure 6C,D). The network module was configured to include a minimum of 40 genes, and a dynamic cutting method was employed to identify diverse gene modules. Modules with high similarity were subsequently merged, resulting in the formation of five distinct gene modules. The relationship between these modules was analyzed (Figure 6E). The results indicate a correlation between MEblack, MEgrey, and AS, with the highest correlation. Next, we intersected the genes that were differentially expressed with the ones associated with AS progression. The findings revealed 48 DEGs about AS progression (Figure 6F).

To delve deeper into the regulation of genes related to gut microbiota, we conducted FMT therapy on AS mice. Furthermore, we examined the changes in gene expression in arterial tissues by utilizing high‐throughput sequencing technology. The results indicated the downregulation of 91 genes in the FMT group, whereas 10 genes were upregulated (Figure 6G). The target genes involved in the metabolism of TMAO and their regulation were obtained from the gutMGene database (Table S3). Next, we performed an intersection analysis of the key differential genes associated with AS progression, genes involved in microbiome regulation, and TMAO target genes. We found that Sirt1 plays a key role in the effect of TMAO on AS progression (Figure 6H). We conducted additional investigations into the expression of Sirt1. The results revealed a downregulation of Sirt1 expression in the AS group, whereas the FMT group exhibited an upregulation of Sirt1 expression (Figure 6I,J).

In summary, these findings suggest that TMAO may regulate the progression of AS by modulating Sirt1.

### 3.7 Impact of TMAO on vascular smooth muscle cell proliferation, adhesion, and inflammatory response

VSMCs are the primary cell types in the aorta and most arteries. In a healthy state, blood vessels maintain the stability of their structure and function. However, in the event of blood vessel damage or exposure to adverse environmental factors, smooth muscle cells undergo a transition from a contracting phenotype to a synthesizing phenotype. As a result of the increase in their adhesion and proliferation abilities, arterial plaques are formed.^15^ According to relevant literature, α‐SMA is a specific marker molecule for VSMCs.^33^

Our metabolomics analysis revealed that the gut microbiota plays a crucial role in the metabolism of arterial plaque (AS). TMAO was identified as a key metabolite. TMAO is a compound produced by the gut microbiota by metabolizing specific nutrients, including phospholipids, choline, and carnitine. Several studies have demonstrated that TMAO is implicated in the regulation of inflammation lipid metabolism, as well as exerting both indirect and direct effects on vasodilation and vasoconstriction.^9^

**FIGURE 7:**
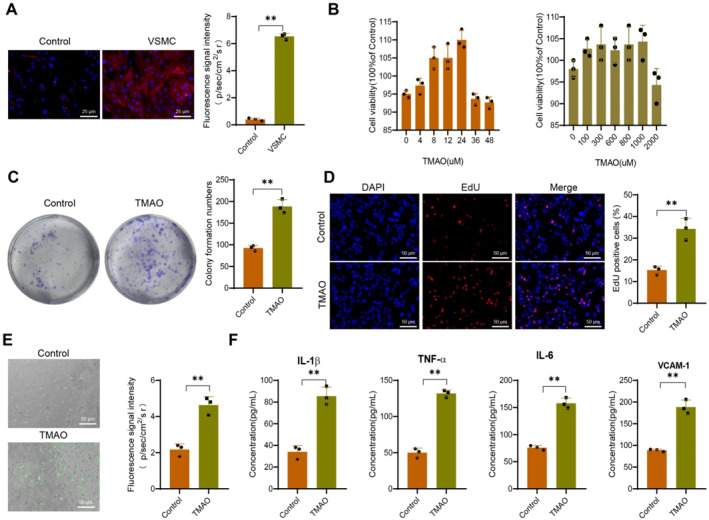
Trimethylamine‐N‐oxide TMAO regulates the expression of Sirt1 and VCAM‐1 in mouse vascular smooth muscle cells (VSMCs). (A) Immunofluorescence experiment screening α‐smooth muscle actin positive VSMCs, with the right side showing quantitative statistics. (B) In treating VSMCs with different concentrations of TMAO (100–2000 μM) for 24 h, cell viability was detected using MTT assay and quantification analysis of cell viability. Treatment of VSMCs with TMAO (600 μM) for 4–48 h, cell viability detected using MTT assay and quantification analysis of cell viability. (C) Colony formation assay showing colony numbers in control and TMAO groups, with the right side showing quantitative statistics. (D) EdU assay to detect proliferation ability of control and TMAO groups (scale bar = 50 μm), with the right side showing quantitative statistics. (E) Representative images from fluorescence microscopy showing the adhesion ability of control and TMAO groups (scale bar = 50 μm), with the right side showing quantitative statistics. (F) Enzyme‐linked immunosorbent assay kit detection of inflammatory factor levels in VSMCs. The Control group refers to untreated VSMC. Data shown are mean ± SD, *n* = 6 for each group, from three independent experiments. **p < 0.01, using one‐way analysis of variance and *t*‐test. MTT, 3‐(4,5‐dimethylthiazol‐2‐yl)‐2,5‐diphenyltetrazolium bromide; VCAM‐1, vascular cell adhesion molecule‐1.

To examine the potential impact of TMAO on cellular functional changes in VSMCs, we initially conducted cell immunofluorescence assays to screen for α‐SMA positive VSMC cells. Compared to the control group, the VSMC group exhibited a noteworthy increase in α‐SMA expression (Figure 7A). To assess the potential cytotoxic effects of TMAO on primary mouse VSMCs, based on relevant literature, we exposed the cells to TMAO concentrations ranging from 100 to 2000 μM for 24 h. Alternatively, we exposed the cells to a concentration of 600 μM TMAO for a specific duration, followed by an MTT assay to measure cell viability.^34^ ^,^ ^58^ Figure 7B demonstrates that within the concentration range of 100–2000 μM, TMAO did not affect cell viability, indicating the absence of cytotoxic effects under the specified conditions. We further verified the effect of TMAO on AS using a colony formation assay. The results showed that compared with the control group, cells in the TMAO group exhibited significantly enhanced colony‐forming ability (Figure 7C). The proliferation ability of cells in the TMAO group was enhanced after treatment with TMAO, as evidenced by the EdU assay, compared to the control group (Figure 7D). In our adhesion experiments, we observed that TMAO stimulation enhanced the adhesion of bone marrow‐derived macrophages (BMDM) to VSMCs (Figure 7E). This result suggests that TMAO could potentially enhance the proliferation and adhesion capabilities of VSMCs. The levels of inflammation‐related factors in the smooth muscle cell inflammation response were determined using an ELISA kit. The results demonstrated an increase in the levels of inflammatory factors IL‐1β, TNF‐α, IL‐6, and vascular cell adhesion molecule‐1 (VCAM‐1) in the TMAO group compared to the control group (Figure 7F).

In conclusion, the findings indicate that TMAO impacts the proliferation and adhesion of VSMCs, which might be closely associated with the upregulation of the inflammatory response.

### 3.8 Inhibition of Sirt1 expression by TMAO promotes cell proliferation, adhesion, and inflammatory responses in VSMCs

**FIGURE 8:**
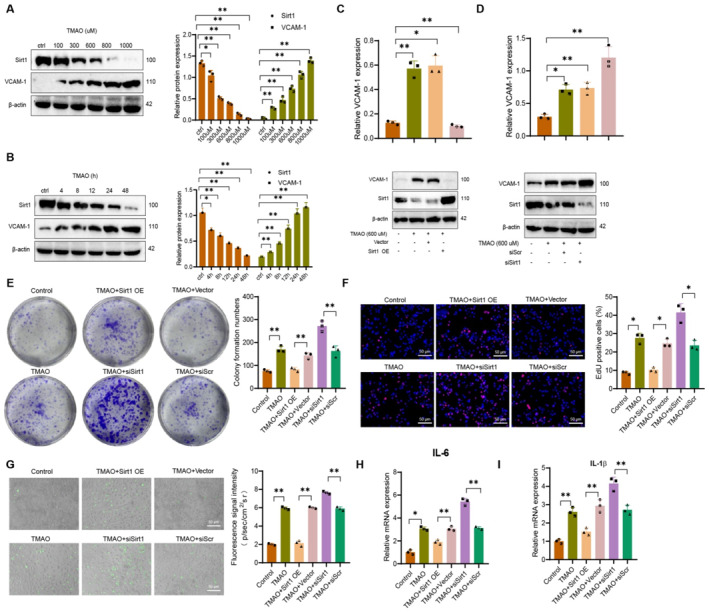
Effects of Sirt1 on TMAO‐induced expression of VCAM‐1, related inflammatory factors, and cell function in vascular smooth muscle cells (VSMCs). (A) Western blot analysis of protein expression levels of VCAM‐1 and Sirt1 in VSMCs after treatment with specified concentrations of trimethylamine‐N‐oxide TMAO for 24 h β‐Actin was used as a reference. (B) Western blot analysis of protein expression levels of VCAM‐1 and Sirt1 in VSMCs after TMAO (600 μM) treatment for specified time points. β‐Actin was used as a reference. (C, D) Transfection of siRNA targeting Sirt1 (siSirt1) and Sirt1 cDNA (Sirt1 OE) in VSMCs, followed by TMAO (600 μM) stimulation for 24 h. Western blot analysis of protein expression levels of VCAM‐1 and Sirt1. β‐Actin was used as a reference. (E) Colony formation assay showing colony numbers in control, TMAO, TMAO + Sirt1 OE, TMAO + siSirt1, TMAO + Vector, and TMAO + siScr groups, with the right side showing quantitative statistics. (F) EdU assay to detect proliferation ability of control, TMAO, TMAO + Sirt1 OE, TMAO + siSirt1, TMAO + Vector and TMAO + siScr groups (scale bar = 50 μm), with the right side showing quantitative statistics. (G) Representative images from fluorescence microscopy showing the adhesion ability of control, TMAO, TMAO + Sirt1 OE, TMAO + siSirt1, TMAO + Vector and TMAO + siScr groups (scale bar = 50 μm), with the right side showing quantitative statistics. (H, I) Real‐time quantitative reverse transcription PCR analysis of IL‐6, IL‐1β mRNA expression levels in VSMCs from different groups. Data shown are mean ± SD, *n* = 6 for each group, from three independent experiments. *p < 0.05, **p < 0.01, using one‐way analysis of variance and *t*‐test. PCR, polymerase chain reaction; VCAM‐1, vascular cell adhesion molecule‐1.

Previous studies have demonstrated a close association between TMAO and inflammatory reactions, whereas inflammatory conditions impact VSMCs.^15^ Our transcriptome analysis revealed that TMAO may play a role in AS progression by regulating the core target gene Sirt1. To explore the alterations in related factors following TMAO treatment, we dose‐dependently induced the expression of Sirt1 and VCAM‐1 in primary cultured mouse VSMCs using TMAO at concentrations ranging from 100 to 1000 μM (Figure 8A). Furthermore, the expression levels of Sirt1 and VCAM‐1 in mouse VSMCs treated with a concentration of 600 μM TMAO exhibited a time‐dependent trend (Figure 8B). In conclusion, these findings suggest that TMAO could potentially stimulate the upregulation of inflammation‐related factors in smooth muscle cells during the AS process. In VSMCs, the expression changes of Sirt1 and VCAM‐1 induced by TMAO show opposite trends.

To investigate the involvement of Sirt1 in TMAO‐induced expression of VCAM‐1 in primary mouse VSMCs, we observed that overexpression of Sirt1 suppressed VCAM‐1 expression induced by TMAO, upon transfection with Sirt1 cDNA plasmid. However, overexpression of an empty vector did not impact VCAM‐1 expression (Figure 8C). Lowering the expression level of Sirt1 RNA increased VCAM‐1 levels in VSMCs after TMAO induction. In contrast, removing the empty vector siScr had no impact on the expression of VCAM‐1 (Figure 8D).

We also found that under TMAO stimulation, treating cells with siRNA targeting Sirt1 significantly increased their colony formation and proliferation abilities; although overexpression of Sirt1 could significantly inhibit this effect, overexpression of Vector had no significant effect (Figure 8E,F). In TMAO stimulation, BMDMs demonstrate a noticeable increase in adhesion to VSMC. Compared to the TMAO + siScr group, siRNA targeting Sirt1 enhances this adhesive effect. In contrast, the overexpression of Sirt1 through plasmid transfection can inhibit the adhesion ability of BMDM to VSMC.

Conversely, the overexpression of Vector does not possess this inhibitory ability (Figure 8G). Additional experimental results demonstrate that transfection of siRNA specific to Sirt1 leads to an increase in the mRNA expression levels of pro‐inflammatory cytokines IL‐6 and IL‐1β induced by TMAO. Conversely, the overexpression of Sirt1 reduces the mRNA expression levels of these two pro‐inflammatory cytokines induced by TMAO (Figure 8H,I). In conclusion, these findings demonstrate that Sirt1 plays a crucial role in the expression of VCAM‐1 and pro‐inflammatory cytokines in TMAO‐induced VSMCs.

The results mentioned above indicate that TMAO inhibits the expression of Sirt1, resulting in the promotion of cell proliferation, adhesion, and the expression of VCAM‐1 and inflammatory cytokines in VSMCs.

### 3.9 Sirt1 modulates vascular smooth muscle cell inflammatory response and AS progression through SM22α and NF‐kB signaling pathway

SM22α is a myosin‐binding protein found in smooth muscle cells. Its expression is downregulated during the progression of AS. It primarily regulates the inflammatory response of smooth muscle cells and promotes the development of AS via the reactive oxygen species (ROS)‐mediated NF‐kB signaling pathway.^59^

**FIGURE 9:**
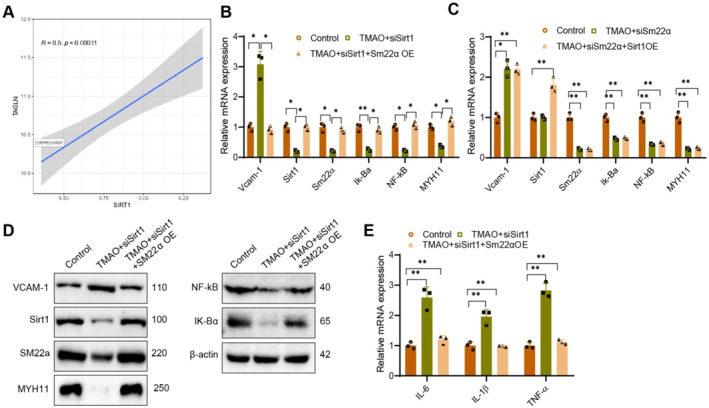
Sirt1 regulates TMAO‐induced expression of VCAM‐1 and inflammatory reaction mediated by SM22α. (A) Analysis of correlation expression between Sirt1 and SM22α (gene name TAGLN) based on transcriptomic data. (B, C) Real‐time quantitative reverse transcription PCR (RT‐qPCR) analysis of Vcam‐1, Sirt1, Sm22α, MYH11, Ik‐Bα, and NF‐kB mRNA expression levels in vascular smooth muscle cells (VSMCs) from different groups. (D) Western blot analysis of protein expression levels of VCAM‐1, Sirt1, SM22α, MYH11, Ik‐Bα, and NF‐kB in arterial tissues. β‐Actin was used as a reference. (E) RT‐qPCR analysis of IL‐6, IL‐1β, and TNF‐α mRNA expression levels in VSMCs from different groups. Data shown are mean ± SD, *n* = 6 for each group, from three independent experiments. *p < 0.05, **p < 0.01, using one‐way analysis of variance and *t*‐test. PCR, polymerase chain reaction; VCAM‐1, vascular cell adhesion molecule‐1.

To delve deeper into the precise mechanism by which Sirt1 triggers inflammation in TMAO‐induced VSMCs, the initial step involved analyzing its expression using transcriptomic data analysis. The results demonstrate a positive correlation between the expression of Sirt1 and SM22α (also known as TAGLN) (Figure 9A). To investigate the correlation between Sirt1, SM22α, and MYH11 expressions, we performed cellular experiments. The RT‐qPCR detection results revealed a decrease in the expression of Sirt1 and SM22α in the TMAO + siSirt1 group compared to the control group. Moreover, the proteins Ik‐Bα and NF‐kB in the NF‐kB signaling pathway were downregulated.

Additionally, there was an increase in the expression of the adhesion factor Vcam‐1.

Nevertheless, the occurrence of this trend was noticeably mitigated with the replenishment of SM22α (Figure 9B). Moreover, we performed cellular experiments involving treatment with TMAO + siSM22α. Compared to the control group, the TMAO + siSM22α group showed no significant change in Sirt1 expression, but exhibited significantly decreased expression of SM22α, MYH11, IκBα, and NF‐κB, along with increased VCAM‐1 expression. Following Sirt1 overexpression, no significant changes were observed in SM22α, MYH11, IκBα, NF‐κB, or VCAM‐1 expression (Figure 9C). The results of RT‐qPCR were confirmed by Western blot analysis. In comparison to the control group, the TMAO + siSirt1 group exhibited suppressed expression of Sirt1, SM22α, MYH11, NF‐kB, and IK‐Bα in VSMCs while promoting the expression of the adhesion factor VCAM‐1 in VSMCs. Following the cell treatment of TMAO + siSirt1 + SM22α overexpression, the expression of adhesion factor VCAM‐1 decreased, whereas the expression of Sirt1, SM22α, MYH11, NF‐kB, and IK‐Bα increased (Figure 9D). Using RT‐qPCR, we detected the expression of inflammatory factors in VSMC. Our results show that compared to the control group; the TMAO + siSirt1 group exhibited an upregulation in the expression of inflammatory factors IL‐6, IL‐1β, and TNF‐α. However, these inflammatory markers were significantly downregulated in the TMAO + siSirt1 + SM22α‐OE group compared to the TMAO + siSirt1 group (Figure 9E). Generally, the bioinformatics results align with the experimental findings, demonstrating a positive correlation between the expression levels of Sirt1 and SM22α. It is plausible that Sirt1 influences the development of AS by modulating the expression of SM22α.

In conclusion, our findings suggest that Sirt1 may trigger an inflammatory response in VSMCs by modulating the NF‐kB signaling pathway via SM22α. This mechanism consequently influences the progression of AS.

### 3.10 Sirt1 overexpression mitigates AS progression and inflammation by modulating SM22α and NF‐kB signaling in AS mice

Moreover, we subsequently conducted animal experiments to further validate the influence of Sirt1 expression on the progression of AS. Similarly, we utilized CRISPR and hybridization methods to create Sirt1 knock‐in AS mice. After 12 weeks of feeding, we collected relevant tissues from HFD to evaluate the progression of AS.

**FIGURE 10:**
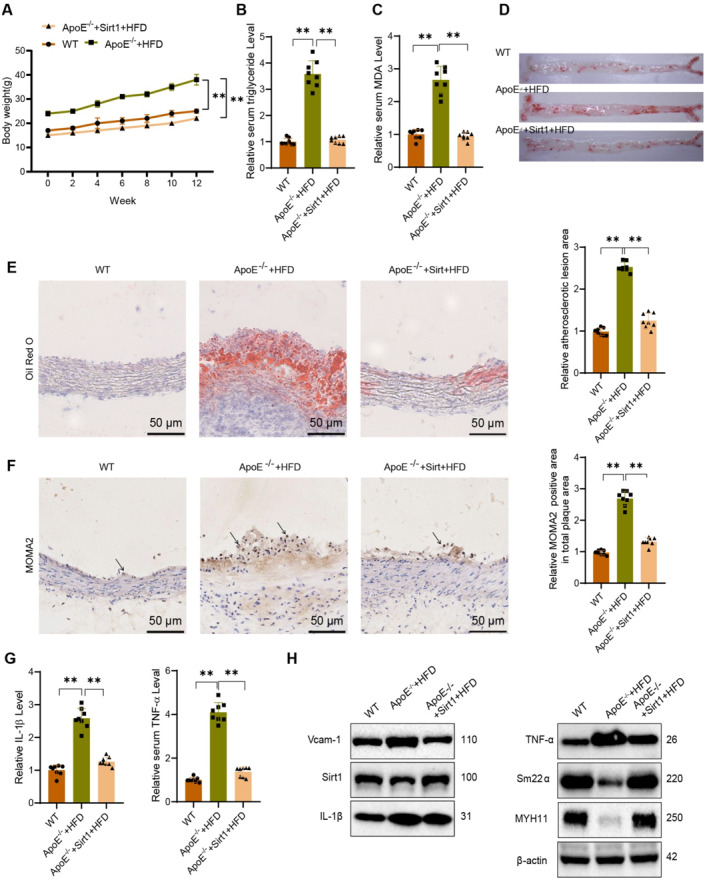
The impact of Sirt1 gene editing on the progression of atherosclerosis (AS) in AS mice. (A) Growth curve of body weight in each treatment group of mice. (B, C) Serum levels of triglyceride and malondialdehyde in each group of mice. (D) Representative images of aortic lesions in each group of mice stained with Oil Red O. (E) Quantification of tissue lesion levels in aortic root sections stained with Oil Red O, scale bar = 50 μm. (F) Immunohistochemical detection of macrophage infiltration in aortic tissue, scale bar = 50 μm, with black arrows indicating the locations of infiltrated macrophages. (G) Serum levels of IL‐1β and TNF‐α in each group of mice, as detected by ELISA. (H) Expression of AS‐related proteins in the aorta, determined by Western blot; *n* = 10 per group; **p < 0.01; Significance not marked refers to comparison with the control group.

The test results indicate that the progression of AS was inhibited in Sirt1 knock‐in mice (ApoE^−/−^ + Sirt1 + HFD) compared to the mice in the AS model group (ApoE^−/−^ + HFD). Specific manifestations include slowed weight gain (Figure 10A), decreased levels of serum TG and the oxidative stress marker MDA (Figure 10B,C), a reduction in the number and volume of aortic plaques as observed through oil red O staining (Figure 10D), a decrease in the area of aortic root tissue lesions (Figure 10E), a decrease in the number of monocyte‐macrophages in the arteries (Figure 10F), and a reduction in tissue inflammation levels (Figure 10G). Immunoblotting results revealed a reduction in the expression of VCAM‐1, IL‐1β, and IK‐Bα in the mouse model's aortic tissue upon Sirt1 overexpression. The expression of Sirt1, MYH11, and SM22α was also noticeably increased (Figure 10H), suggesting that Sirt1 could modulate the SM22α‐mediated NF‐kB signaling pathway, leading to VSMC inflammation inhibition and regulation of AS progression.

The findings suggest that the upregulation of Sirt1 could hinder the progression of AS. This effect could be attained by regulating the expression of SM22α and mediating the NF‐kB signaling pathway to suppress the inflammatory response of VCAM.

## 4 DISCUSSION

Over the past few decades, a substantial amount of research has focused on AS, primarily due to its role as a leading cause of cardiovascular diseases, including coronary artery disease.^60^, ^61^ Previous studies have comprehensively explored the effects of factors such as blood lipids, inflammatory responses, and endothelial dysfunction.^62^ In recent years, the relationship between gut microbiota and various diseases has gained increasing attention with advancements in microbiomics.^63^, ^64^, ^65^ Furthermore, this study takes an additional step by examining the detailed association between the gut microbiota metabolite TMAO and AS.

Significant differences in the gut microbiota were observed between AS and normal mice, particularly in Bacteroides and Firmicutes. This finding aligns with earlier research that also identified variations in these two bacterial types among cardiovascular disease patients.^10^, ^66^, ^67^ These results suggest a potential link between the gut microbiota and the progression of AS. This study provides explicit evidence of the crucial role of TMAO in AS, as demonstrated through metabolomic analysis. Previous research has suggested an association between TMAO and an increased risk of cardiovascular diseases. However, our study uniquely uncovers a novel correlation between TMAO, SM22α, and SIRT1, shedding light on the molecular mechanisms underlying TMAO's impact on AS. SIRT1, a deacetylase nuclear protein, has been previously implicated in various diseases, especially those related to aging and cancer.^68^, ^69^, ^70^ The connection between SIRT1, the gut microbiota metabolite TMAO, and the inflammatory response of smooth muscle cells represents a new finding in this study. This research not only advances our understanding of SIRT1's functions but also identifies new potential targets for the treatment of AS.

The findings of this study hold importance for clinical medicine. If additional confirmation is obtained regarding the potential of TMAO to trigger AS, it could be feasible to develop more accurate prevention and treatment strategies for AS patients through the regulation of gut microbiota or TMAO levels in the future. This treatment option may be especially beneficial for high‐risk individuals with poorly controlled regular blood lipid levels, providing them with a new and innovative approach to treatment. This study has elucidated the direct relationship between gut microbiota metabolites and the onset of AS in contrast to prior research. Although previous studies have suggested a link between gut microbiota and cardiovascular health, this study provides experimental evidence elucidating the molecular mechanism by which TMAO contributes to AS.^71^ These findings enhance the current understanding of AS pathogenesis and identify specific molecular targets that may be leveraged in the development of microbiota‐based therapeutic strategies.

**FIGURE 11:**
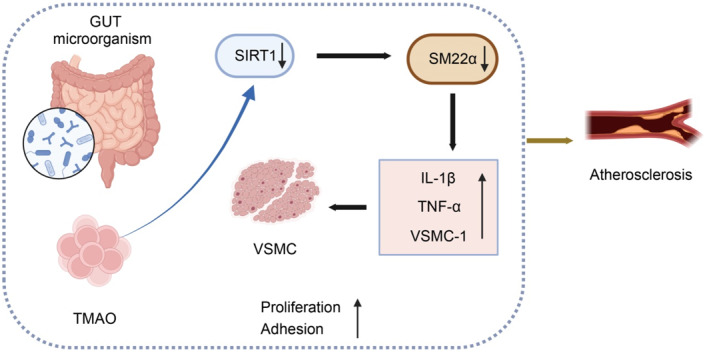
Molecular mechanism of intestinal microbial metabolite trimethylamine‐N‐oxide regulating SM22α‐mediated smooth muscle cell inflammatory response and promoting atherosclerosis.

Based on the experiments above, we could tentatively draw the following conclusion: during the progression of AS, alterations in the gut microbiota lead to a considerable elevation in the metabolic activity of the crucial metabolite TMAO, which inhibits the expression of SIRT1. Consequently, this downregulates SM22α and induces inflammation in smooth muscle cells, thereby advancing the progression of AS (Figure 11).

This study elucidates the mechanism by which the gut microbiota‐derived metabolite TMAO promotes AS by modulating smooth muscle cell inflammation through SM22α. This conclusion was supported by multivariate analyses. The results advance our understanding of the mechanistic link between gut microbiota and cardiovascular pathology and provide a foundation for the development of microbiota‐targeted therapeutic strategies. The observed inhibition of SIRT1 by TMAO may enhance SM22α expression and facilitate AS progression. Accordingly, future therapeutic approaches may focus on modulating TMAO or its upstream metabolic pathways to manage cardiovascular diseases such as AS. The primary objective of this study was to investigate the impact of a HFD in the ApoE^−/−^ mouse model. WT mice were used as baseline controls, which is a widely accepted approach to distinguish dietary effects from genetic predisposition. ApoE^−/−^ mice fed with an HFD closely mimic the pathological features of AS, whereas WT mice help evaluate the influence of HFD alone. This design allows for a clear assessment of diet–genotype interactions. Although ApoE^−/−^ mice that were fed a normal diet were not included due to logistical limitations, we acknowledge this as a study limitation and intend to address it in future research. It is worth noting that previous studies have similarly adopted WT mice as controls in comparable contexts.^72^, ^73^ Ongoing research into gut microbiota–host interactions has the potential to facilitate individualized cardiovascular risk assessment through microbiota profiling. This could support preventive strategies and personalized interventions, including dietary and lifestyle modifications tailored to microbiome composition.

Although mouse models are extensively used in disease research, species‐specific differences in physiology and metabolism limit direct extrapolation to humans. Therefore, the current findings require further validation in clinical settings. Moreover, the relationship between gut microbiota and host health is multifactorial. Although this study focused on the TMAO–SM22α axis, additional microbial metabolites and host signaling pathways are likely involved. Unidentified confounding variables may also have influenced the results. Future research should aim to broaden the investigation of gut microbiota metabolites across diverse cardiovascular conditions to inform the development of targeted therapies. In particular, therapeutic interventions targeting TMAO and its regulatory pathways could hold promise for AS management. Further mechanistic studies are warranted to delineate the molecular interactions underlying TMAO‐mediated SM22α regulation, ultimately contributing to the rational design of microbiota‐based treatments.

## AUTHOR CONTRIBUTIONS

Yajuan Yin, Mei Wei, and Xiufang Jiang conceived and designed the study. Mei Liu, Xiaocui Shi, and Xiao Zhang performed the experiments. Le Wang, Gang Liu, and Mingqi Zheng analyzed the data. Yajuan Yin and Fangfang Ma wrote the manuscript. All authors reviewed and approved the final version of the manuscript.

## CONFLICT OF INTEREST STATEMENT

The authors declare no conflicts of interest.

## ETHICS STATEMENT

All experiments involving mice were approved by the Animal Ethics Committee of The First Hospital of Hebei Medical University.

## Supporting information

Supporting Information S1

## Data Availability

All data generated or analyzed during this study are available from the corresponding author upon reasonable request. For inquiries, please contact Dr. Fangfang Ma at mafangfang@hebmu.edu.cn.
