# Supplementary material for: Gut microbiota‐derived trimethylamine‐N‐oxide inhibits SIRT1 to regulate SM22α‐mediated smooth muscle cell inflammation and promote atherosclerosis progression
Source: J Cell Commun Signal. 2025 Jun 6;19(2):e70021. doi: 10.1002/ccs3.70021 (PMC12143968; doi:10.1002/ccs3.70021)
Supplement: Supplementary file 1 — Supporting Information S1 [file CCS3-19-e70021-s001.docx]

**
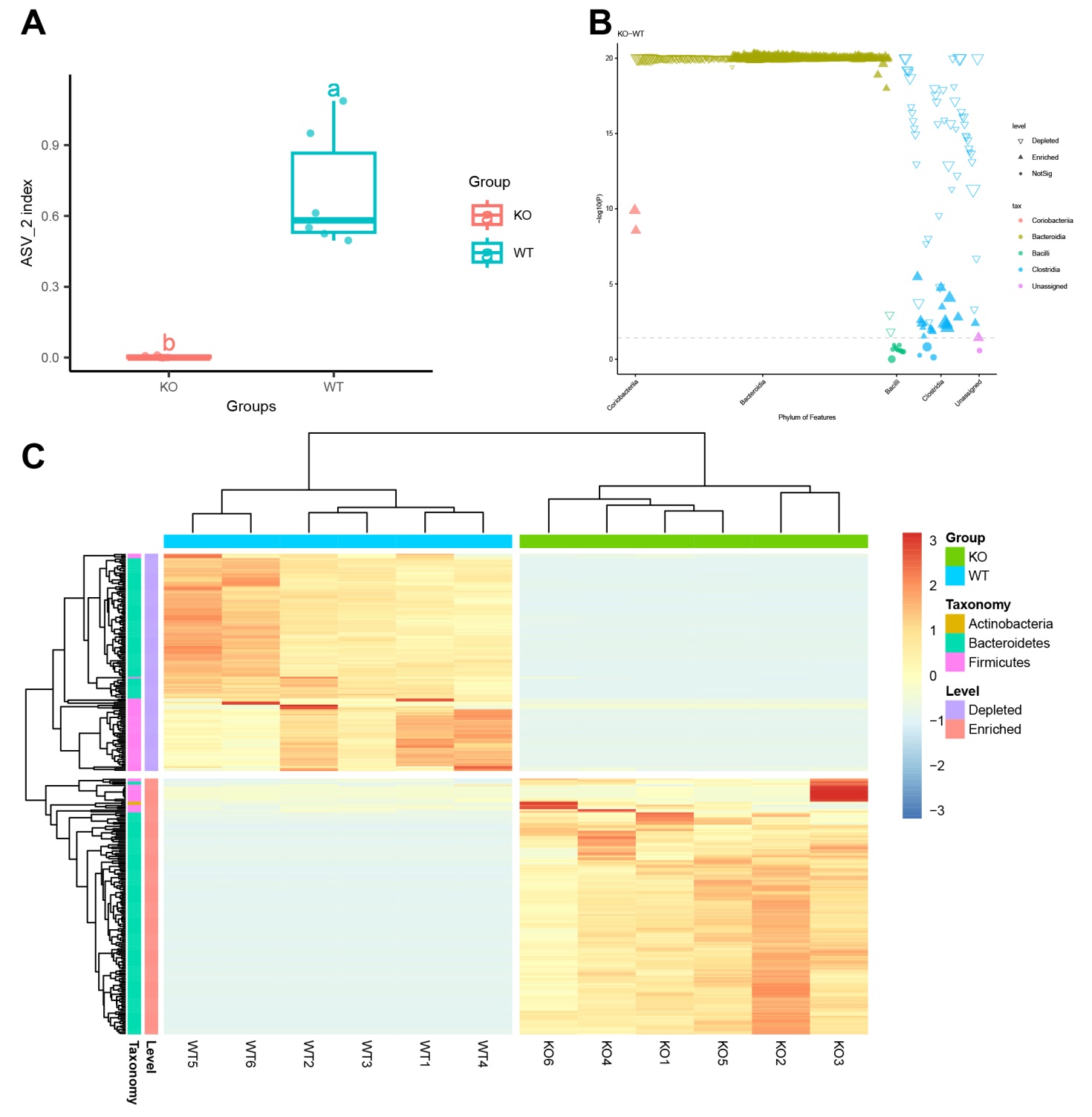
**

**Figure S1. Comparison of Intestinal Microbial Abundance in Fecal Samples of KO Group and WT Group.**

Note: (A) Column chart comparing the abundance of intestinal microbiota between the KO group and the WT group; (B) Manhattan plot comparing the abundance difference of intestinal microbiota between the KO group and the WT group; (C) Heatmap comparing the abundance difference of intestinal microbiota between the KO group and the WT group at the phylum level, with different colors representing different microbial groups at the phylum level; a and b indicate statistical differences between the two groups (*P* < 0.05); KO group, n=6; WT group, n=6.

**
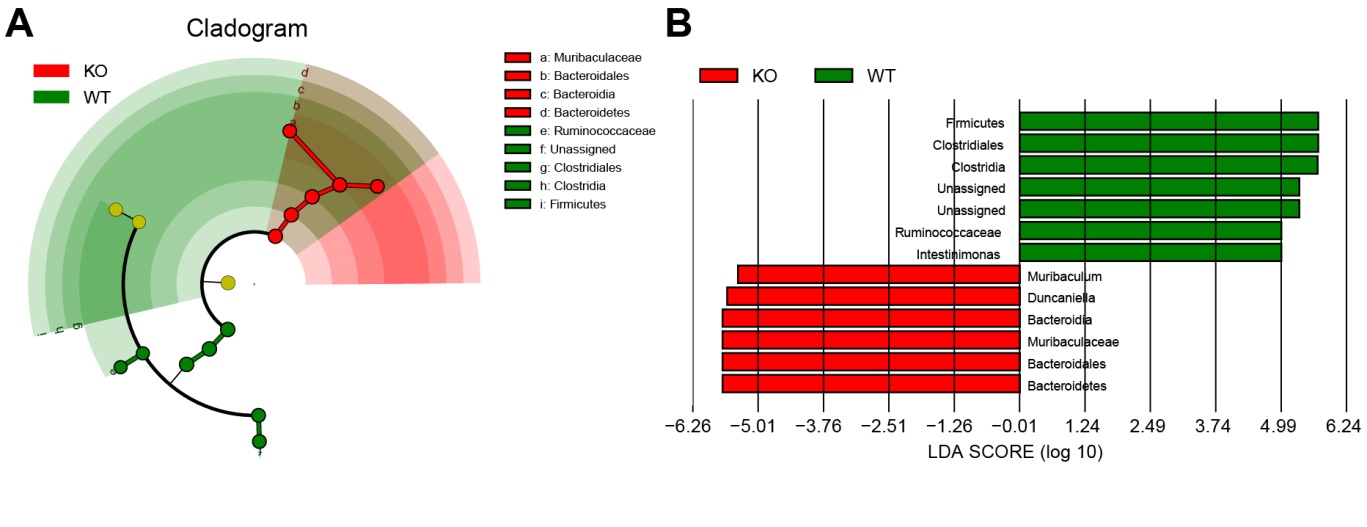
**

**Figure S2. Differential Composition of Intestinal Microbiota at the Genus Level in the KO Group and WT Group Analyzed by LEfSe.**

Note: (A) Radial tree diagram of species abundance of intestinal microbiota in the KO group and the WT group, with concentric circles representing classification levels from kingdom to genus and the diameter representing the relative abundance; yellow nodes represent species with no difference, green nodes represent microbial groups with higher abundance in the WT group, and red nodes represent microbial groups with higher abundance in the KO group; (B) Histogram of LDA values distribution for species abundance in the KO group and the WT group, with green bars indicating microbial groups with higher abundance in the WT group and red bars indicating microbial groups with higher abundance in the KO group; KO group, n=6; WT group, n=6.

**Table S1. The detail of product**

| **Name** | **Cat.** | **Species** | **Dilution ratio** | **Manufacturer** | **Country** |
| --- | --- | --- | --- | --- | --- |
| VCAM-1 | MA5-31965 | Mouse | 1: 500 | Thermo Fisher | USA |
| Sirt1 | PA5-17074 | Mouse | 1: 500 | Thermo Fisher | USA |
| SM22α | PA5-29767 | Mouse | 1: 1000 | Thermo Fisher | USA |
| Ik-Bα | MA5-15132 | Mouse | 1: 1000 | Thermo Fisher | USA |
| TNF-α- | MA5-32618 | Mouse | 1: 500 | Thermo Fisher | USA |
| MYH11 | 14-6400-82 | Mouse | 1：500 | Thermo Fisher | USA |
| β-actin | MA1-140 | Mouse | 1: 5000 | Sigma-Aldrich | USA |

**Table S2. The primer sequence of RT-qPCR**

| **Gene** | **Primer (Mouse)** |
| --- | --- |
| TNF-α | F 5'- ACCCTCACACTCACAAACCA-3' |
|  | R 5'- ATAGCAAATCGGCTGACGGT-3' |
| IL-1β | F 5'- AAATGCCACCTTTTGACAGTGA-3' |
|  | R 5'- TCATATGGGTCCGACAGCAC-3' |
| VCAM-1 | F 5'- GCCCACTAAACGCGAAGGT-3' |
|  | R 5'- ACTGGGTAAATGTCTGGAGCC-3' |
| Sirt1 | F 5'- CGGCTACCGAGGTCCATATAC -3' |
|  | R 5'- CTGCAACCTGCTCCAAGGTA -3 |
| IL-6 | F 5'- CACTTCACAAGTCGGAGGCT -3' |
|  | R 5'- GCCACTCCTTCTGTGACTCC -3 |
| MYH11 | F 5'- GAAAGACAGCAGCATCACGG-3' |
|  | R 5'- CGGCGAGCAGGTAGTAGAAG-3' |
| β-actin | F 5'- CGATATCGCTGCGCTGGTC -3' |
|  | R 5'- ATGGCTACGTACATGGCTGG -3' |

Note: F stands for forward; R stands for reverse.

**Table S3. The metabolic target gene of TMAO and its regulation were obtained from gutMGene database**

| Human / Mouse | Metabolite (ID) | Gene (ID) | Alteration | Throughput | PMID |
| --- | --- | --- | --- | --- | --- |
| mouse | Trimethylamine oxide | Sirt1 (93759) | Reduced | low-throughput | 29325896 |
| mouse | Trimethylamine oxide | Nox4 (50490) | Elevated | low-throughput | 28942145 |
| mouse | Trimethylamine oxide | Tnf (21926) | Elevated | low-throughput | 28942145 |
| mouse | Trimethylamine oxide | Il1b (16176) | Elevated | low-throughput | 28942145 |
| mouse | Trimethylamine oxide | Myh7 (140781) | Elevated | low-throughput | 30068915 |
| mouse | Trimethylamine oxide | Nppa (230899) | Elevated | low-throughput | 30068915 |
| mouse | Trimethylamine oxide | Il1b (16176) | Elevated | low-throughput | 32862276 |
| mouse | Trimethylamine oxide | Il6 (16193) | Elevated | low-throughput | 32862276 |
| mouse | Trimethylamine oxide | Tnf (21926) | Elevated | low-throughput | 32862276 |
| mouse | Trimethylamine oxide | Il10 (16153) | Elevated | low-throughput | 32862276 |
| mouse | Trimethylamine oxide | Nfkb1 (18033) | Elevated | low-throughput | 32862276 |
| mouse | Trimethylamine oxide | Lcn2 (16819) | Elevated | low-throughput | 32862276 |
